# Understanding polypharmacy for people receiving home care services: a scoping review of the evidence

**DOI:** 10.1093/ageing/afaf031

**Published:** 2025-02-19

**Authors:** Radin Karimi, Anna Robinson-Barella, Vanessa Davey, David R Sinclair, Barbara Hanratty, Adam Todd

**Affiliations:** School of Pharmacy, Faculty of Medical Sciences, Newcastle University, Newcastle upon Tyne, Tyne and Wear, UK; National Institute for Health and Care Research—Newcastle Patient Safety Research Collaborative, Newcastle University, Newcastle upon Tyne, Tyne and Wear, UK; School of Pharmacy, Faculty of Medical Sciences, Newcastle University, Newcastle upon Tyne, Tyne and Wear, UK; National Institute for Health and Care Research—Newcastle Patient Safety Research Collaborative, Newcastle University, Newcastle upon Tyne, Tyne and Wear, UK; Population Health Sciences Institute, Faculty of Medical Sciences, Newcastle University, Campus for Ageing and Vitality, Newcastle upon Tyne, Tyne and Wear, UK; Population Health Sciences Institute, Faculty of Medical Sciences, Newcastle University, Campus for Ageing and Vitality, Newcastle upon Tyne, Tyne and Wear, UK; Population Health Sciences Institute, Faculty of Medical Sciences, Newcastle University, Newcastle upon Tyne, Tyne and Wear, UK; Newcastle Biomedical Research Building, Population Health Sciences Institute, Faculty of Medical Sciences, Newcastle University, Campus for Ageing and Vitality, Newcastle upon Tyne, Tyne and Wear, UK; National Institute for Health and Care Research—Newcastle Patient Safety Research Collaborative, Newcastle University, Newcastle upon Tyne, Tyne and Wear, UK; Population Health Sciences Institute, Faculty of Medical Sciences, Newcastle University, Campus for Ageing and Vitality, Newcastle upon Tyne, Tyne and Wear, UK; National Institute for Health and Care Research—Newcastle Patient Safety Research Collaborative, Newcastle University, Newcastle upon Tyne, Tyne and Wear, UK; Population Health Sciences Institute, Faculty of Medical Sciences, Newcastle University, Campus for Ageing and Vitality, Newcastle upon Tyne, Tyne and Wear, UK; School of Pharmacy, Newcastle University, Newcastle upon Tyne, Tyne and Wear, UK

**Keywords:** multiple medications, domiciliary care, home health care, inappropriate prescribing, homecare older people

## Abstract

**Background:**

Polypharmacy, defined as the concurrent use of five or more medications, is common amongst older adults receiving home care services. The relationship between home care and polypharmacy may be critical to older people’s health, but there is little research on this topic.

**Objective:**

To understand the extent and type of evidence on individuals receiving home care services and experiencing polypharmacy.

**Methods:**

This review followed the Preferred Reporting of Items for Systematic Reviews and Meta-Analyses extension for scoping reviews. Three databases (MEDLINE, Embase, CINAHL) were systematically searched (December 2023) to identify studies with adult participants experiencing polypharmacy and receiving home care.

**Results:**

Twenty-three studies were included. For individuals receiving home care services, the studies reported on the following: (i) prevalence of polypharmacy, (ii) interventions to reduce inappropriate polypharmacy, (iii) perceived role of home care workers, (iv) assessment of health literacy in individuals experiencing polypharmacy, and (v) factors associated with polypharmacy and potentially inappropriate medications (PIMs). Polypharmacy and PIMs were found to be associated with older age, female sex, increased frailty, living alone, poor economic situation and inaccuracies within medical records. Improved appropriateness of prescribing can be achieved through interprofessional interventions, efficient use of home care workers and improved health literacy.

**Conclusion:**

This review highlights research on the extent of polypharmacy in home care and ways to address it. Whilst there are suggestions for enhancing medication quality, key gaps remain in research into the experiences of care staff and recipients in managing medications and polypharmacy, which should be addressed.

## Key Points

- This review is the first to explore polypharmacy for individuals receiving home care services, which provide support with activities of daily living, including personal care.
- Polypharmacy is common amongst older adults, and those living with frailty and multiple long-term conditions. This is the population most likely to need homecare support.
- Home care workers are not health care professionals but are well placed to help older adults with their medications.
- Interprofessional interventions, and enhanced health literacy can potentially reduce inappropriate polypharmacy.
- Future studies should seek to explore the experiences people providing, and receiving, home care, particularly in the context of polypharmacy and managing medications.

## Introduction

Multimorbidity, the occurrence of two or more diseases or conditions [1], is prevalent in older adults and is frequently accompanied by polypharmacy (concurrent use of five or more medications) [2, 3]. Polypharmacy can be ‘appropriate’ when medications align with evidence-based care for complex conditions [4], or ‘problematic’ when risks outweigh the intended benefits [5]. Problematic polypharmacy, resulted from over-prescribing or prescription of potentially inappropriate medications (PIMs) [5, 6], poses a significant financial burden on healthcare systems and is associated with negative health outcomes, including developing adverse drug reactions (ADRs), drug–drug interactions, medication errors, healthcare utilisation and increased rates of mortality [6].

Polypharmacy has been extensively studied across various health and social care settings. In United Kingdom (UK) care homes, researchers have reported a polypharmacy prevalence rate of 62% [7]. Factors associated with polypharmacy include increasing age, lower wealth, multimorbidity and obesity [8]. One area where polypharmacy has seldom been explored is in individuals who are receiving social care support in their own homes—a type of support known as home or domiciliary care. In the UK, there are ~12 500 officially registered home care organisations offering services to an estimated 959 000 people, predominantly older people [9], and that number is expected to increase in the future [10]. There is currently no global definition for home care, but it typically involves help with tasks such as personal care, administration of medications and other individual or household activities [11, 12]. Using home care providers to provide medication support adds an additional layer of complexity in already fragmented health and social care systems. This could potentially increase the risk of patient safety events, such as medication errors. Moreover, there are international differences in the provision of home care [13], with implications for medication support. Countries such as Germany, predominantly employ healthcare professionals (e.g. registered nurses) [14], whilst others, such as the UK, have no degree-level educational requirements [15], and <10% of home care staff having a nursing degree [14].

People who receive support with daily activities are also likely to have one or multiple long-term health conditions that may require treatment with a range of medications. As older people prefer to remain in their own homes as they age, any adverse reactions or consequences of complex medication regimens will be experienced there. It will be essential to understand the specific challenges and opportunities for optimising polypharmacy management in the home care setting. No published or ‘in-progress’ systematic reviews or scoping reviews have specifically sought to investigate polypharmacy in individuals receiving home care services. Previous reviews have explored the extent of drug-related problems [16], medication management [17], and adverse events experienced by individuals receiving home care services [18], with only one identifying polypharmacy as a key concern [17]. Conducting a scoping review that is focused on polypharmacy in the home care setting will improve understanding of the specific challenges faced by individuals experiencing polypharmacy, as well as the potential impact of interventions designed to address these challenges. The aim of this scoping review was to explore the extent and type of evidence in relation to individuals receiving home care services experiencing polypharmacy.

## Methods

### Protocol and registration

The review followed the Preferred Reporting of Items for Systematic Reviews and Meta-Analyses—extension for scoping reviews (PRISMA-ScR) framework (see Appendix 1, Supplementary Data) [19]. The scoping review protocol was registered with Open Science Framework (OSF) (registration: nu3w6) [20].

### Eligibility criteria and search strategy

The review sought published material exploring the extent of polypharmacy in the home care setting, in addition to studies examining the effectiveness of interventions in reducing polypharmacy in individuals receiving home care services. Eligible studies for inclusion consisted of adults (any age) taking five or more medications, receiving home care services or staff providing home care to adults experiencing polypharmacy. Home care was defined as a service that enables individuals with physical, mental or cognitive impairment to live at home [12, 21]. Studies that did not provide an explicit definition of home care, but reported on ‘home care patients’ or ‘patients receiving home care services’, were discussed by the research team to reach a consensus decision on inclusion.

Randomised controlled trials, non-randomised controlled trials, before and after studies, interrupted time-series studies, prospective and retrospective cohort studies, case–control studies, analytical cross-sectional studies, case series, individual case reports and descriptive cross-sectional studies were all eligible for inclusion. Studies published in English, irrespective of their geographical location and year of publication, were included. Articles were excluded if they were not conducted in home care settings or populations that were outside the scope of our study (e.g. conducted in home health care, end-of-life care, hospice care and home medical care), were not in English, did not report polypharmacy, or involved people aged under 18 years.

A pilot search of MEDLINE, Embase and CINAHL was undertaken to identify relevant articles on the topic. Moreover, online databases such as Cochrane library and PROSPERO were explored to ensure the review question had not been addressed in any published or ‘in-progress’ reviews. A broader search strategy was developed under the headings of ‘polypharmacy’ and ‘home care’ for MEDLINE (Ovid), Embase (Ovid) and CINAHL (EBSCO) (see Appendix 2, Supplementary Data) (December 2023). The reference list of all included sources of evidence was then hand searched to identify additional studies.

### Selection of sources of evidence

Following the search, all identified citations were collated and uploaded to EndNote 21, then Rayyan QCRI, to remove duplicates. Titles and abstracts were screened by two independent reviewers for assessment against the inclusion criteria for the review. The full text articles were assessed in detail against the inclusion criteria by two independent reviewers (RK and VD). Any disagreements between the reviewers at each stage of the selection process were resolved through discussion; if consensus could not be reached, a discussion was had with the wider review team (AR-B, DS, BH and AT). The Cohen’s kappa statistic was used to test interrater reliability.

### Charting the data

A data extraction tool developed by the authors (Appendix 3, Supplementary Data) was used to report the date and country of study’s conduct, definition of home care, definition of polypharmacy, age, sample size, setting and key findings. As this was a scoping review, no quality appraisal of the included studies was conducted [22].

### Collating, summarising and synthesis of results

The extracted information was tabulated, and a narrative synthesis summarising the findings was undertaken. Included studies were categorised according to their objectives, methodologies and outcomes. Trends and evidence gaps within the literature were then identified.

## Results

**Figure 1:**
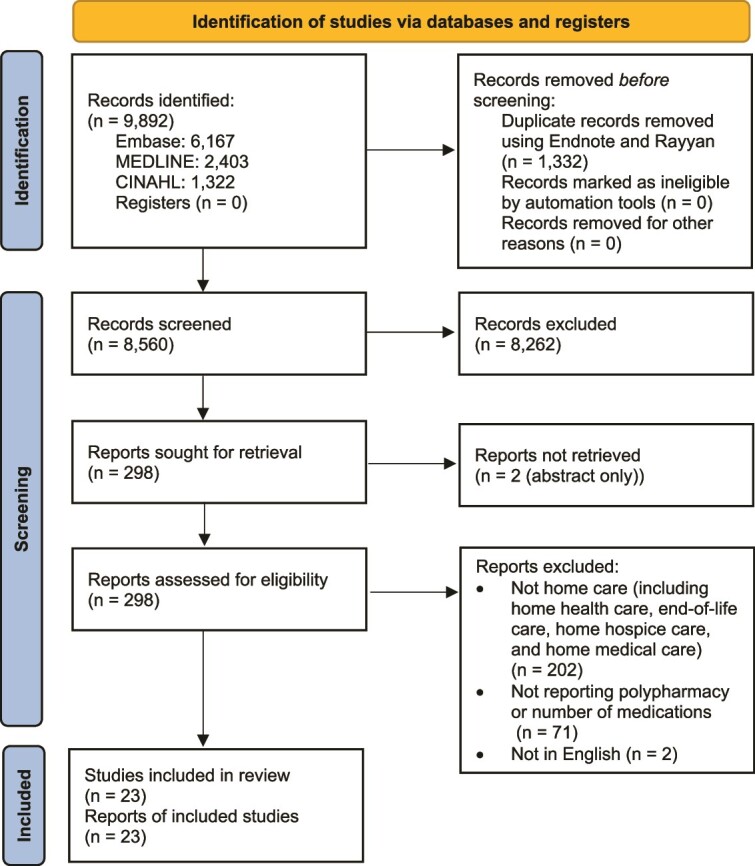
PRISMA flow diagram for scoping review detailing the search process [23].

The search retrieved 8560 articles for title and abstract screening after de-duplication. Upon checking 296 full-text articles and two abstracts, 23 studies were included in the review (Figure 1). Interrater reliability between the authors was perfect, with a Cohen’s Kappa of 1.0. Studies were excluded at the full text stage for the following reasons: (i) not reporting polypharmacy or the use of five or more medications by an individual, (ii) not reporting home care services, (iii) studies not focusing on home care setting (e.g. home health care, end-of-life care, hospice care and home medical care), and (iv) not being in the English language.

### Characteristics of the included studies

**Figure 2:**
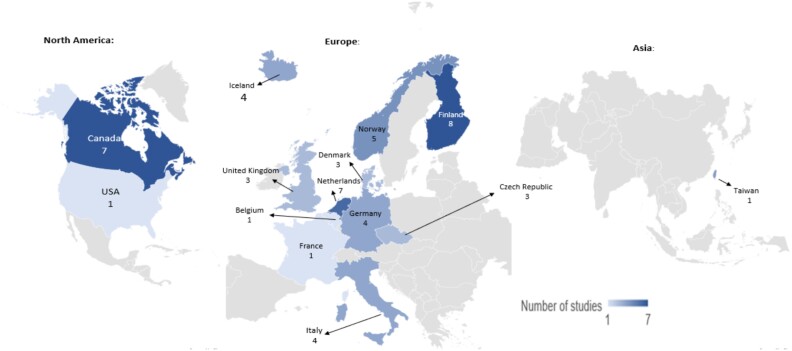
Map of the geographical locations of included studies with regards to number of studies per territory.

Included studies were published from 2000 to 2021, with 14 of the 23 studies being published after 2015. Studies were conducted across 14 different countries, including Canada [24–30], Finland [31–38], Netherlands [31, 33, 34, 37, 39–41], Norway [31, 33, 37, 42, 43], Germany [31, 34, 37, 44], Italy [31, 33, 34, 37] and Iceland [31, 33, 34, 37] (Figure 2). The study designs included cross-sectional studies, retrospective cohort studies, longitudinal cohort studies and randomised controlled trials. Eight studies included participants aged 65 years and older [27, 29, 32–34, 44–46], three studies included participants aged 75 years and over [36, 40, 43], whilst two studies included participants aged 18 years and over [25, 30]. Six studies reported mean age of participants, ranging from 71.5 to 83 years [24, 26, 28, 31, 37, 38]. The population size reported in the studies ranged from 45 in a pilot study [38], to 438,114 in a retrospective cohort study [25]. A summary of the characteristics of the included studies is illustrated in Appendix 4.

### Definitions of home care

Six studies provided a definition or offered any explanation of how home care services were defined [24, 28, 29, 32, 39, 47]. Three studies were from Canada, in which home care was defined as ‘a service that helps individuals of all ages (birth to extreme old age) live in their home and community, by providing support with medical, nursing, physiotherapy, social and therapeutic treatments, and assistance with activities of daily living’ [24, 28, 29]. The two studies from Netherlands defined home care as ‘a service that provides care in individuals’ own homes by nurses of varying educational levels (such as nurse aides, registered nurses and licenced practical nurses), with the aim of assisting and educating individuals of all ages in activities of daily living including help with getting dressed and bathing, pharmacotherapy and medication intake, wound care and disease treatment to promote well-being and greater independence, in order to prevent hospital admission or admission to long-term care organisations’ [39, 40]. A similar definition was used by the study from Finland; defining home care as ‘services that provide support with activities of daily living, home nursing, rehabilitation and end-of-life care’ [32].

Reviewing the topic at the centre of the included studies resulted in identification of the following groups: (i) prevalence of polypharmacy in individuals receiving home care services, (ii) interventions to reduce inappropriate polypharmacy, (iii) perceived role of home care workers in management of medication, (iv) assessment of health literacy in individuals experiencing polypharmacy and (v) factors associated with polypharmacy and PIMs in individuals receiving home care services.

### Prevalence of polypharmacy in individuals receiving home care services

Out of 23 included studies, 16 studies reported the prevalence of polypharmacy or number of participants taking five or more medications. This ranged from 41.1% (reported by Doran *et al*. [25] for one region of the study where polypharmacy was defined as ≥9 medications) to 89.5% (reported by Schneider *et al*., [44] where polypharmacy was defined as ≥5 medications), with eight studies reporting prevalence rates of 80% or higher [27–29, 31, 35, 42–44]. Moreover, six studies reported on excessive polypharmacy (≥10 medications) [34–36, 43, 44, 46], which ranged from 21.0% [46] to 54.9% [36]. Overall, polypharmacy was found to be common in the home care setting (see Appendix 4). Whilst no studies specifically examined the prevalence of problematic polypharmacy, several investigated the prescription of PIMs [33, 42, 43, 45]. The reported prevalence of PIMs in home care settings ranged from 13.8% [43] to 27.0% [42]; one study found an overall prevalence of 19.8%, with significant regional variation; the Czech Republic had the highest prevalence at 41.1%, compared to just 5.8% in Denmark [33]. In the UK, the prevalence of PIMs in home care settings was 14.2% [33].

### Interventions to reduce inappropriate polypharmacy

One study reported the effects of an intervention to reduce inappropriate polypharmacy and improve medication quality in the context of home care. The study, by Auvinen *et al*., examined the effects of an interprofessional medication assessment on medication quality amongst home care patients [32]. The interprofessional team consisted of a pharmacist, physician and registered nurse who reviewed patients’ medications and health conditions, and implemented recommendations regarding prevention of drug–drug interactions, risks of drug-induced renal impairment, medication-related risk loads and PIMs [32]. During the six-month follow up period, the intervention reduced inappropriate polypharmacy by decreasing the risk of anticholinergic burden, ADRs (e.g. renal impairment, bleeding and constipation) and the use of PIMs [32].

### Perceived role of home care workers in management of medication

Three studies explored the role of home care workers. The study, by Dijkstra *et al.*, explored medication adherence support provided by home care nurses [39], and identified the most common support provided to individuals receiving home care services which included ‘noticing when I don’t take medications as prescribed’, ‘helping to find solutions to overcome problems with using medications’, ‘helping with taking medications’ and ‘explaining the importance of taking medications at the right moment’ [39]. This study also suggested that individuals receiving home care services had negative experiences of care, which stemmed from inadequate timing of home visits, rushed visits from providers and insufficient expertise of home care workers regarding side effects and medication administration [39]. Moreover, Sino *et al*. explored home care workers’ ability to detect potential ADRs using a standardised observation list [40]. Home care workers reported signs or symptoms in 80% of patients [40]. Similarly, in the study by Dimitrow *et al.*, 82% of staff’s drug-related problem recommendations were validated by a geriatrician, with ADR-related symptoms being the key risk factors [38].

### Assessment of health literacy in individuals experiencing polypharmacy

Health literacy refers to possession of the appropriate knowledge, skills and understanding to obtain, comprehend, evaluate and navigate health and social care information and services [48, 49]. Three studies explored aspects of health literacy for individuals experiencing polypharmacy receiving home care services. Medication knowledge and the ability to take medication were reviewed, where it was shown that increasing the number of prescribed medications was associated with decreased medication knowledge by individuals taking six or more medications [29]. A significant number of participants lacked the knowledge and skills to independently manage their medication, with a considerable proportion of participants being unable to state the names of their medications and having problems with opening packaging [41]. Moreover, in a study by Sun *et al.*, which explored the impact of therapeutic self-care on the types and frequency of adverse events, low therapeutic self-care ability was associated with an increase in adverse events such as falls, unplanned hospital visits and unintended weight loss [30].

### Factors associated with polypharmacy and potentially inappropriate medication

Several studies explored factors associated with polypharmacy and PIMs in individuals receiving home care services. PIM use was found to be associated with a patient’s poor economic situation, polypharmacy, anxiolytic drug use, age 85 years and over, living alone, female sex and depression [33, 42]. Polypharmacy was associated with chronic disease, multimorbidity, female sex, old age, dyspnoea and falls [34]. In a study by Huang *et al.*, polypharmacy and excessive polypharmacy were associated with lower risk of mortality comparing to non-polypharmacy [46], whilst Larsen *et al.* report that polypharmacy was associated with increased frailty [27]. Moreover, studies found polypharmacy to be associated with increased hospitalisation due to cardiovascular events [28], reduced therapeutic self-care ability [30], and a decreased probability of dementia-related hospitalisation [35]. Similar trends were seen regarding the association of excessive polypharmacy and increased rates of inaccuracies within patient medical records [36].

**Figure 3:**
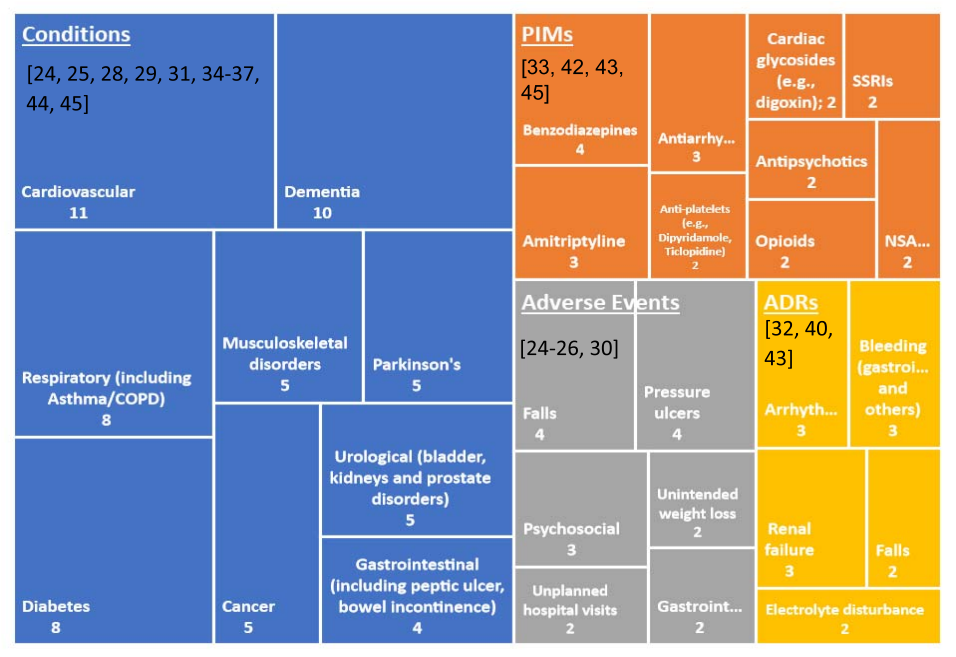
Treemap of the most common conditions, PIMs, adverse events and ADRs as reported by studies. Numbers 2–11 represent the number of studies. Numbers in brackets are citations.

A treemap highlighting the most common conditions, PIMs, adverse events and ADRs reported in individuals receiving home care services is shown in Figure 3. Common conditions included dementia, cardiovascular and respiratory diseases and diabetes. PIMs (as reported in the studies [33, 42, 43, 45]) included benzodiazepines, tricyclic antidepressants, antiarrhythmics and opioids. Common adverse events experienced by individuals receiving home care services, included falls, pressure ulcers, psychosocial (e.g. delirium and suicide thoughts) and unintended weight loss, whilst frequent ADRs included arrhythmias, bleeding, renal failure and falls.

## Discussion

Polypharmacy is a major public health issue [5]. The management of polypharmacy in the home care setting will be an important future issue, as an increasing proportion of the ageing population prefer to receive care within their own homes, rather than move into care homes [9, 50]. This scoping review has identified five categories of evidence on polypharmacy and home care services. Existing research describes the extent of polypharmacy and associated factors, potential interventions, the perceived role of home care staff, and health literacy amongst care recipients. Notable gaps in the literature include exploration of the experiences people providing, and receiving, home care, particularly in relation to polypharmacy and medication management.

In this review, polypharmacy was found to be common in individuals receiving home care services, ranging from 41.1% to 89.5% across studies. Individuals receiving home care services found interventions provided by the home care workers helpful in adhering to their medication regimens. However, inadequate timing of home visits, and insufficient expertise of home care workers regarding administration and side effects of medications were highlighted as potential areas for improvement. Given the challenges faced by individuals with multimorbidity, advanced medical treatments at home, and the risks associated with handling medications by lone home care workers, it is crucial that appropriate training is in place to minimise medication errors and risk of harm to individuals receiving home care services [51, 52]. Medication training for home care nurses has been shown to considerably reduce the number of medication errors, and increase their awareness of polypharmacy and the necessity of deprescribing within the home care setting [47, 53, 54]. Whether training for home care workers who are not nurses would be feasible, safe or acceptable is unknown. There is currently a gap in the literature regarding the perceptions of home care and support workers in relation to medication management issues.

In this review, individuals with polypharmacy were found to be at risk of experiencing ADRs and adverse events such as falls. Around a third of people aged 65 and over fall at least once a year [55]. Home care recipients are likely to be at greater than average risk and vulnerable to adverse consequences, including increased mortality [55]. The cause of falls in the home care setting can be multifactorial; however, it can be directly linked to common PIMs reported in this setting, such as benzodiazepines and amitriptyline [56]. Benzodiazepines and amitriptyline have been shown to significantly increase the risk of falls for several reasons, including the effects of increasing anticholinergic burden, cognitive impairment, muscle relaxation and central nervous system depression [57]. Pharmacist-led medication reviews in residential aged care settings and primary care have been shown to considerably reduce the prescription of PIMs and promote appropriate polypharmacy [58, 59]. In this review, an interprofessional medication assessment intervention carried out by a team of pharmacists, physicians and nurses was shown to improve medication quality by reducing the anticholinergic burden and prescription of PIMs in individuals receiving home care services. In addition to medication reviews, interventions such as home safety assessments to identify potential hazards, exercise programmes to improve strength and balance, and educational programmes regarding the use of assistive devices (e.g. canes and walkers) have been effective in prevention of falls in individuals receiving home health care [60, 61]. Home care workers are not in a position to deprescribe, but they are uniquely positioned to observe care recipients daily, monitor for signs of ADRs or missed doses, and escalate concerns to healthcare professionals. Enhancing communication pathways between home care workers and healthcare professionals could ensure that potential issues are addressed promptly, thereby improving medication safety.

Studies in this review reported that a significant number of individuals receiving home care services do not have the ability to independently manage their own medications. Taking a higher number of medications was linked to reduced knowledge and understanding of medications. Individuals who lacked knowledge about their medications and the ability to manage their treatment were more likely to experience adverse events, such as falls and unplanned hospital visits. Low levels of health literacy have been shown to cause challenges regarding medication management, ultimately resulting in increased rates of polypharmacy [62, 63]. Digital health interventions, such as using apps to access and apply health information [64], as well as health literacy training [63], have been shown to improve health literacy for people in the home care setting. Further research could explore the feasibility of implementing digital health interventions to improve health literacy in individuals experiencing polypharmacy in the home care setting, and the appropriate involvement of home care workers.

This is the first review to explore the nature of existing evidence in relation to polypharmacy in individuals receiving home care services—specifically defined as a service that enables individuals to live at home, within their own communities. There are important differences in the definitions of home care used by previous reviews compared to this review, which included referring to home care as services that address the treatment of health conditions in the patient’s home [17], or services that enable patients to live at home with the support of professional caregivers (mostly nursing professionals) [16]. This review recognises that home care can also be provided for purposes of rehabilitative and supportive care. By defining home care as a service that enables people with mental, physical and cognitive to live at home, this review provides a clear overview of evidence regarding challenges faced by individuals experiencing polypharmacy, in addition to possible interventions that could positively impact medication management in this setting.

Our findings have several practise and policy implications. Firstly, the different services described as home care highlight the need for the adoption of a standard definition. This may help to ensure that the heterogeneity and breadth of clients and services are appreciated when planning and delivering medication related interventions [65]. Identification of common PIMs may be an important preventive initiative, as they are predictors of inappropriate polypharmacy and adverse events. An adequately resourced home care service could be well placed to have a role in the detection of ADRs, which calls for interventions such as medication reviews to improve medication safety in this setting. In the UK, pharmacists have recently been given greater roles in care homes [66]. However, the situation in home care is more complex, involving multiple prescribers from different primary care organisations. Future research could explore optimal models for pharmacist involvement in home care, and the role of preventative strategies aimed at care staff, families and care recipients, to minimise the risk of adverse events.

This scoping review adds to the literature by identifying the most common PIMs and adverse events experienced by individuals with polypharmacy who receive home care services. We also highlight challenges faced by these individuals, emphasising the role that home care workers may play in the safer management of polypharmacy in this setting, juxtaposed by concerns over inadequate levels of expertise and time to do so.

Limitations of this review include the requirements of studies being published in the English language, only using three databases and lack of universal definition for home care and polypharmacy. Studies exploring home health care, end-of-life care, home hospice care and home medical care were not included as they did not fit our definition of home care. Broadening the definition of home care to encompass all variations of home care across different countries could result potentially in the identification of more studies. Furthermore, extending the definition of polypharmacy, for instance, to the concurrent use of two or more medications, could result in the inclusion of more studies.

## Conclusion

This review provides a comprehensive overview of evidence on polypharmacy in individuals receiving home care services. Whilst suggestions such as improving health literacy, better utilisation of home care workers, and interprofessional medication assessment interventions have the potential to positively impact polypharmacy, their feasibility needs to be explored further. Key gaps identified through this review around the experiences of care staff and recipients in managing medications and polypharmacy need to be addressed by future studies.

## Supplementary Material

aa-24-2206-File002_afaf031(1)
