# Supplementary material for: Understanding polypharmacy for people receiving home care services: a scoping review of the evidence
Source: Age Ageing. 2025 Feb 19;54(2):afaf031. doi: 10.1093/ageing/afaf031 (PMC11837856; doi:10.1093/ageing/afaf031)
Supplement: aa-24-2206-File002_afaf031(1) [file aa-24-2206-file002_afaf031(1).docx]

Understanding polypharmacy for people receiving home care services: a scoping review of the evidence

**Supplementary Data**

Table of Contents

[Appendix 1. Preferred Reporting Items for Systematic reviews and Meta-Analyses extension for Scoping Reviews (PRISMA-ScR) Checklist 1](#_Toc188031729)

[Appendix 2. Search strategy 3](#_Toc188031730)

[Appendix 3. Data extraction tool 6](#_Toc188031731)

Appendix 4. Table of characteristics of the included studies ……………………………..7

### Appendix 1. Preferred Reporting Items for Systematic reviews and Meta-Analyses extension for Scoping Reviews (PRISMA-ScR) Checklist

| **SECTION** | **ITEM** | **PRISMA-ScR CHECKLIST ITEM** | **REPORTED ON PAGE #** |
| --- | --- | --- | --- |
| **TITLE** | | | |
| Title | 1 | Identify the report as a scoping review. | #1 |
| **ABSTRACT** | | | |
| Structured summary | 2 | Provide a structured summary that includes (as applicable): background, objectives, eligibility criteria, sources of evidence, charting methods, results, and conclusions that relate to the review questions and objectives. | #1 |
| **INTRODUCTION** | | | |
| Rationale | 3 | Describe the rationale for the review in the context of what is already known. Explain why the review questions/objectives lend themselves to a scoping review approach. | #2 |
| Objectives | 4 | Provide an explicit statement of the questions and objectives being addressed with reference to their key elements (e.g., population or participants, concepts, and context) or other relevant key elements used to conceptualize the review questions and/or objectives. | #2 |
| **METHODS** | | | |
| Protocol and registration | 5 | Indicate whether a review protocol exists; state if and where it can be accessed (e.g., a Web address); and if available, provide registration information, including the registration number. | #2 |
| Eligibility criteria | 6 | Specify characteristics of the sources of evidence used as eligibility criteria (e.g., years considered, language, and publication status), and provide a rationale. | #2-3 |
| Information sources* | 7 | Describe all information sources in the search (e.g., databases with dates of coverage and contact with authors to identify additional sources), as well as the date the most recent search was executed. | #2-3 |
| Search | 8 | Present the full electronic search strategy for at least 1 database, including any limits used, such that it could be repeated. | #2-3 |
| Selection of sources of evidence† | 9 | State the process for selecting sources of evidence (i.e., screening and eligibility) included in the scoping review. | 3 |
| Data charting process‡ | 10 | Describe the methods of charting data from the included sources of evidence (e.g., calibrated forms or forms that have been tested by the team before their use, and whether data charting was done independently or in duplicate) and any processes for obtaining and confirming data from investigators. | #3 |
| Data items | 11 | List and define all variables for which data were sought and any assumptions and simplifications made. | #3 |
| Critical appraisal of individual sources of evidence§ | 12 | If done, provide a rationale for conducting a critical appraisal of included sources of evidence; describe the methods used and how this information was used in any data synthesis (if appropriate). | #3 |
| Synthesis of results | 13 | Describe the methods of handling and summarizing the data that were charted. | #3 |
| **RESULTS** | | | |
| Selection of sources of evidence | 14 | Give numbers of sources of evidence screened, assessed for eligibility, and included in the review, with reasons for exclusions at each stage, ideally using a flow diagram. | #3-4 |
| Characteristics of sources of evidence | 15 | For each source of evidence, present characteristics for which data were charted and provide the citations. | #3-4 |
| Critical appraisal within sources of evidence | 16 | If done, present data on critical appraisal of included sources of evidence (see item 12). | Not applicable |
| Results of individual sources of evidence | 17 | For each included source of evidence, present the relevant data that were charted that relate to the review questions and objectives. | #4-6 |
| Synthesis of results | 18 | Summarize and/or present the charting results as they relate to the review questions and objectives. | #4-6 |
| **DISCUSSION** | | | |
| Summary of evidence | 19 | Summarize the main results (including an overview of concepts, themes, and types of evidence available), link to the review questions and objectives, and consider the relevance to key groups. | #6-7 |
| Limitations | 20 | Discuss the limitations of the scoping review process. | #8 |
| Conclusions | 21 | Provide a general interpretation of the results with respect to the review questions and objectives, as well as potential implications and/or next steps. | #8 |
| **FUNDING** | | | |
| Funding | 22 | Describe sources of funding for the included sources of evidence, as well as sources of funding for the scoping review. Describe the role of the funders of the scoping review. | #8 |

JBI = Joanna Briggs Institute; PRISMA-ScR = Preferred Reporting Items for Systematic reviews and Meta-Analyses extension for Scoping Reviews.

* Where *sources of evidence* (see second footnote) are compiled from, such as bibliographic databases, social media platforms, and Web sites.

† A more inclusive/heterogeneous term used to account for the different types of evidence or data sources (e.g., quantitative and/or qualitative research, expert opinion, and policy documents) that may be eligible in a scoping review as opposed to only studies. This is not to be confused with *information sources* (see first footnote).

‡ The frameworks by Arksey and O’Malley (6) and Levac and colleagues (7) and the JBI guidance (4, 5) refer to the process of data extraction in a scoping review as data charting*.*

§ The process of systematically examining research evidence to assess its validity, results, and relevance before using it to inform a decision. This term is used for items 12 and 19 instead of "risk of bias" (which is more applicable to systematic reviews of interventions) to include and acknowledge the various sources of evidence that may be used in a scoping review (e.g., quantitative and/or qualitative research, expert opinion, and policy document).

*From:* Tricco AC, Lillie E, Zarin W, O'Brien KK, Colquhoun H, Levac D, et al. PRISMA Extension for Scoping Reviews (PRISMAScR): Checklist and Explanation. Ann Intern Med. 2018;169:467–473. [doi: 10.7326/M18-0850](http://annals.org/aim/fullarticle/2700389/prisma-extension-scoping-reviews-prisma-scr-checklist-explanation).

### Appendix 2: Search strategy

Ovid platforms search strategy:

| **Polypharmacy** | **Homecare** |
| --- | --- |
| Polypharmacy/ | ("home care" or "homecare" or "home caring" or "home help" or "home helps" or "homemaker services" or "homemaker service" or "home maker service" or "home maker services" or "home support" or "home carer" or "home carers" or "home caregivers" or "home caregiver" or "home service" or "home services" or "home assistance").tw. |
| polypharma*.ti,ab. | exp Home Care Services/ |
| Polypharmac*.tw. | Home Care Agencies/ |
| Inappropriate Prescribing/ | "Personal care service*".tw. |
| medication adherence/ | ((Domicili* or shop* or "own home" or "home based" or "in the home" or "in home" or "at home" or hous*) adj2 ("assist*" or "care" or service* or "caring" or "support*")).tw. |
| patient medication knowledge/ | ((care adj2 "individual* home*") or ("care in" adj1 "home*")).tw. |
| (yellow adj2 card*).tw. | (("personal care" and home*) or (home* adj2 assistance) or ("personal assist*" and home*)).tw. |
| (MHRA adj2 safe*).tw. | ((shar* or shelt* or support* or special* or temp*) adj2 (scheme* or hous* or home* or liv* or accommodat*)).tw. |
| ((medication* or medicine* or medical* or meds or drug* or tablet*) adj2 (adherence* or compliance* or aid* or concordance*)).tw. |  |
| ("monitored dosage system*" or MDS).tw. |  |
| ((medication* or medicine* or medical* or meds or drug* or tablet* or blister*) adj2 (pack* or box* or case*)).tw. |  |
| multiple medication*.mp. |  |
| multiple drug*.mp. |  |
| many medication*.mp. |  |
| many medicine*.mp. |  |
| many drug*.mp. |  |
| drug related side effects and adverse reactions/ |  |
| ((incident* or safeguard* or coroner* or medication* or medicine* or medical* or meds or pharmac* or drug* or tablet*) adj2 (report* or system* or audit*)).tw. |  |
| ((medication* or medicine* or medical* or meds or drug* or tablet*) adj2 (order* or deliver* or request*) adj2 (system* or process*)).tw |  |
| ((remov* or dispos* or expire* or waste* or deliver* or collect* or supp* or dispen* or transport*) adj2 (medication* or medicine* or medical* or meds or drug* or tablet*)).tw. |  |

CINAHL search strategy:

| **Polypharmacy** | **Homecare** |
| --- | --- |
| polypharmacy or multiple drugs or medications | MH "Home Health Care+" |
| “inappropriate prescribing medicine” | MH "Home Health Aides" |
| “medication adherence OR medication errors” | MH "Home Care Equipment and Supplies" |
| MH "Medication Errors" | MH "Home Health Care Information Systems" |
| MH "Medication History" | domiciliary care or home care or home nursing |
| MH "Medication Prescribing (Iowa NIC)" | Homecare or home care or home health or homehealth |
| polypharmacy or polymedication or multiple medication or multiple drug | "Personal care" |
| (MH "Polypharmacy") OR (MH "Polypharmacy (Saba CCC)") OR "Polypharmac*" | (MH "Home Health Nurses") OR (MH "Health Services for Older Persons") OR "homecare services OR homecare nursing OR homecare medicine service" |
| (MH "Medication Compliance") OR "((incident* or safeguard* or coroner* or medication* or medicine* or medical* or meds or pharmac* or drug* or tablet*) W2 (report* or system* or audit*))." | (MH "Home Respiratory Care") OR (MH "Home Health Agencies") OR "home care medication safety management OR home care patients OR home care agencies OR domiciliary visit OR home support OR home support service" |
| "((medication* or medicine* or medical* or meds or drug* or tablet*) adj2 (order* or deliver* or request*) W2 (system* or process*)). | "personal care services OR personal care assistant OR home assistance OR home health care OR home based care OR home-based intervention OR home help OR home help service OR home caregiver" |
| (MH "Medication Errors") OR "remov* or dispos* or expire* or waste* or deliver* or collect* or supp* or dispen* or transport*) W2 (medication* or medicine* or medical* or meds or drug* or tablet*" | "("home care" or "homecare" or "home caring" or "home help" or "home helps" or "homemaker services" or "homemaker service" or "home maker service" or "home maker services" or "home support" or "home carer" or "home carers" or "home caregivers" or "home caregiver" or "home service" or "home services" or "home assistance")." |
| ((medication* or medicine* or medical* or meds or drug* or tablet* or blister*) W2 (pack* or box* or case*))." | (Domicili* or shop* or "own home" or "home based" or "in the home" or "in home" or "at home" or hous*) W2 ("assist*" or "care" or service* or "caring" or "support*") |
| "Many medicine* OR Many drug* OR many medication* OR multiple medication* OR Multiple drug*" |  |
| "inappropriate prescribing OR patient medication knowledge OR MHRA W2 safe*" OR (MH "Inappropriate Prescribing") |  |
| (MH "Medication Systems") OR "monitored dosage system OR MDS" |  |

### Appendix 3: Data extraction tool

| Author (Year) | Aim | Setting | Design | Definition of home care | Definition of polypharmacy | Population | Age | Findings |
| --- | --- | --- | --- | --- | --- | --- | --- | --- |
|  |  |  |  |  |  |  |  |  |

| **Appendix 4**: Table of characteristics of the included studies  Author (year) | Aim | Setting | Design | Definition of home care | Definition of polypharmacy | Population | Age | Findings |
| --- | --- | --- | --- | --- | --- | --- | --- | --- |
| Alanen *et al*. (2008) [31] | To ascertain the differences in the use of antipsychotic medications between European home-care sites. | Nine European  countries (Czech Republic, Denmark, Finland, Germany, Iceland, Italy, Netherlands, Norway and United  Kingdom) | Cross-sectional study using Resident Assessment Instrument for Home Care (RAI-HC) questionnaires | Not Reported | Not Reported | 200 | Mean age: 82.2 | - 81.4% of the population were using more than five medications.  - The prevalence of the use of one or more antipsychotics varied widely among the sites studied, from 3.0% in Denmark to 12.4% in Finland. |
| Auvinen *et al.* (2021) [32] | To assess the effects of interprofessional medication assessment on medication quality among home care patients. | Home care settings in Finland | Randomised controlled  study comparing physician-led interprofessional medication assessment and usual care | Finnish home care services include support and assistance in activities of daily living (ADLs), home nursing and physician services, rehabilitation, home hospital services in acute situations, and the end- of-life care. Patients who are regular clients of home care services must have substantial functional limitations in basic and instrumental ADLs. | Not Reported | 512 | ≥65, Mean: 84.15 | - The mean number of all prescription and Over-the-Counter (OTC) counterdrugs taken regularly or as needed was 15 in both study groups; range: 4‒36 in the intervention and 2‒32 in the usual care group. The proportion of patients using 9 or more drugs was 92% in the intervention and 94% in the usual care group. The mean number of regularly taken drugs was 9.2 (range: 2-20) in the intervention and 9.5  (range: 1‒20) in the usual care group. The number and range of drugs taken as needed was 3.5 (0-20) and 3.8 (0-13) in the intervention and usual care groups, respectively.  - The intervention improved the medication quality of home care patients. Risks for renal failure, anticholinergic effects, bleeding, constipation, and the use of PIMs were reduced significantly. |
| Blais *et al*. (2013) [24] | To document the incident rate and types of adverse events (AEs) among home care clients, factors contributing to AEs, and the extent of evidence regarding completion of incident reports for AEs. | Publicly funded home care programmes in Manitoba, Quebec and Nova Scotia, Canada | Retrospective cohort study based on chart review of a random sample of clients discharged from publicly funded home care programmes | Home care includes the provision of healthcare interventions to clients of all ages (birth to extreme old age), for the purposes of providing curative, supportive, palliative and rehabilitation care for acute and long-term illnesses and conditions. | Not Reported | 1200 | Mean age: 71.52 | - 568 clients were taking 4-9 medications with 36 clients experiencing adverse events.  - 301 clients were taking 9 or more medications, out of which 34 clients had experienced adverse events.  - The most frequent AEs were injuries from falls, wound infections, psychosocial, behavioural or mental health problems and adverse outcomes from medication errors.  - Only 17.3% of charts with an AE contained documentation that indicated an incident report was completed. |
| Dijkstra *et al*. (2021) [39] | To describe nurses’ support interventions for medication adherence, and patients’  experiences and desired improvements with home care. | Netherlands | A two-phase study including analysis of questionnaire data and interviews with members of the care panel | Home care in the Netherlands involves care delivered in patients’ homes by nurses of different educational levels, such as registered nurses, licensed practical nurses, and nurse aides. The goal of home care is to assist individuals of all ages (but mainly adults) to improve function and live with greater independence, to promote well-being, and to assist individuals in several therapies, such as pharmacotherapy or activities of daily living so they can remain at home and avoid hospital admission or admission to long-term care organisations. Home care nurses work intra- and interdisciplinary and their roles concerning medication adherence involve observing and addressing non-adherence. | Not Reported | 59 (of which, 14 did the interview) | Median age: 68  (IQR 59–72) | - 37% of the participants were taking 6-10 prescription medications, and 31% were taking more than 10 medications.  - The most received adherence support interventions are ‘’noticing when I don’t take medications as prescribed’’ ‘’helping me to find solutions to overcome problems with using medications”, “helping me with taking medication” (e.g., opening packages), and “explaining the importance of taking medication at the right moment”.  - Twenty-two participants experienced the following as positive: improved self-management of adequate medication taking, a professional patient–nurse relationship to discuss adherence problems, and nurses’ proactive attitude to arrange practical support for medication use.  - Thirteen patients experienced the following as negative: insufficient timing of home visits, rushed appearance of nurses, and insufficient expertise about side effects and taking medication. |
| Dimitrow *et al*. (2018) [38] | To establish the sensitivity of practical nurse administered drug-related problems risk assessment tool compared with geriatrician’s assessment of the medical record. | Finland | Pilot study | Not reported | Not reported | 45 | Mean age: 83 range = 64-96 | - Mean number of regular prescription medications in participants = 9.5 (range = 4-15). Mean number of as needed prescription medications = 2.9 (range = 0-12).  - Symptoms suggestive of adverse drug reactions were the most significant risk predicting factors.  - Practical nurses’ recommendations were valid in 82% of the cases, as appraised by the geriatrician.  - The intervention provided clinically important timely patient information for clinical decision making. |
| Doran *et al.* (2013) [25] | To investigate adverse events in home care, specifically those associated with hospitalisation or detected through the Resident Assessment Instrument for Home Care (RAI-HC). | Nova Scotia, Ontario, British Columbia and the Winnipeg Regional Health  Authority, Canada | Retrospective study design | Not Reported | ≥9 medications | 438,114 | ≥18, Mean age: 72.17 | - The overall incidence rate of adverse events associated with hospitalisation ranged from 6% to 9%.  - The adverse event incident rate determined from RAI-HC was 4%.  - Medication related events (such as accidental poisoning, adverse effect at therapeutic dos, overdose and haemorrhagic disorders due to anticoagulants), injurious fall, other injuries such as burns, contact with heat and hot substances, and accidental drowning) were the most frequent adverse events associated with hospitalisation.  - Polypharmacy was only reported for Nova Scotia, Ontario and Winnipeg region, in which polypharmacy was seen in 41.1%, 49.1% and 42.2% of the participants respectively. |
| Doran *et al.* (2009) [26] | To identify the nature of patient safety problems among Canadian home care clients, using data collected through the RAI-HC assessment problems. | Ontario, Nova Scotia, Winnipeg, Canada | A secondary analysis of data collected through the Canadian home care reporting system | Not Reported | Not Reported | 238,958 | 16.17% <65, 16.87% 65-74, 66.9% ≥75 | - The most prevalent safety risk for home care clients was polypharmacy, followed by decline in physical function.  - Polypharmacy was seen in 72.9% of service users across the three regions.  - 25.6% of clients with polypharmacy were also experiencing cognitive decline.  - Between 1% to 4% clients who had a history of polypharmacy and cognitive decline, had no medication review since their previous RAI-HC assessment. |
| Fialová *et al.* (2005) [33] | To estimate the prevalence and associated factors of potentially inappropriate medication use among elderly home care patients in European countries. | Metropolitan areas of the Czech Republic, Denmark, Finland, Iceland, Italy,  Netherlands, Norway, and the United Kingdom | Retrospective cross-sectional study | Not Reported | ≥6 medications | 2707 | ≥65, Mean: 82.2 | - Potentially inappropriate medication use was associated with patient’s poor economic situation, polypharmacy, anxiolytic drug use and depression.  - Negatively associated factors were age 85 years and older, and living alone. The odds of potentially inappropriate medication use significantly increased with the number of associated factors.  - Polypharmacy was documented in 51% of patients.  - 46.6% of patients with no inappropriate medication use, and 68.8% of patients with inappropriate medication use were experiencing polypharmacy.  - The most common inappropriate medications were Pentoxifylline, Diazepam and Amiodarone. |
| Flaherty *et al*. (2000) [45] | To examine the relation between medication use (number, type, and inappropriateness) and hospitalisation among home care patients older than 65 years. | One Home care agency in St. Louis, USA | A retrospective chart review of persons discharged from a home care agency | Not Reported | No arbitrary number to define polypharmacy. Three different cutoff points were chosen: 5, 7, and 10 | 833 | ≥65,  Mean: 77.5 | -. The Hospitalised group, compared with the self-care or family care group, was taking a higher number of medications, and had a higher percentage of patients taking 7 or more medications (46% vs 26%, p = 0.002) and 10 or more medications (21% vs 10%, p = 0.005), but not 5 or more medications.  - Only three types of medications were more commonly used among patients in the Hospitalised group than among patients in the S/F Care group: clonidine (4.2% vs 1.1%, p 5 .004); mineral supplements (23.8% vs 14.8%, p <5 .003); and metoclopramide (5.8% vs 2.0%, p< 5 .006). The Hospitalised group had a lower percentage of patients taking inappropriate medications than did the S/F Care group (20% vs 27%, p < 5.040), but none of the types of inappropriate medications was used more often in either group.  - The most common inappropriate medications across both groups were Propoxyphene, Amitriptyline and Dipyridamole. |
| Giovannini *et al.* (2018) [34] | To assess the prevalence and factors associated with polypharmacy in a sample of home care patients in Europe. | 38 home care organisations across Belgium, Finland, Germany, Iceland, Italy, and the Netherlands | A cross-sectional analysis | Not Reported | Concurrent use of 5-9 drugs. Excessive polypharmacy: 10 or more drugs | 1873 | ≥65, Mean: 83.5 | - Polypharmacy was observed in 730 (39.0%) home care patients and excessive polypharmacy in 433 (23.1%). As compared with non-polypharmacy, excessive polypharmacy was directly associated with chronic disease but also with female sex, dyspnoea, and falls. An inverse association with excessive polypharmacy was shown for age.  - Home care patients on polypharmacy and excessive polypharmacy presented a higher number of concomitant diseases; more specifically, COPD, heart failure, diabetes, and cancer were more common in these groups than in the non-polypharmacy group. |
| Huang *et al.* (2020) (abstract only) [46] | To examine the association between polypharmacy and the risk of mortality in older people who need home care by adopting the time-varying (quarterly) measures of polypharmacy status. | Taiwan | Retrospective longitudinal cohort study | Not Reported | 5-9 medications.  Excessive polypharmacy: 10 or more drugs | 50,210 | ≥65, Mean: 80.5 | - According to participants’ polypharmacy status in the first quarter, 50.8% of participants were experiencing polypharmacy, and 21.0% of participants were experiencing excessive polypharmacy.  - The generalised estimating equation models indicated that polypharmacy and excessive polypharmacy group had lower risk of 5-year all cause mortality than non-polypharmacy group. Similar results were observed at 1- and 3- years of follow-up. |
| Josendal *et al. (*2020) [42] | To examine the quality of prescribing to Norwegian elderly home care service patients receiving multidose drug dispensing (MDD). | Norway | Cross-sectional study using the medication lists from MDD patients in Norway | Not Reported | Not Reported | 45,593 | >70, Mean: 84.7 | - The mean number of regular medications was 8.2 (median = 8), of which 6.1 (median = 6) were dispensed as MDD.  - The mean number of total prescribed medicines was 10.6 (median = 10). In total 85% used 5 or more medicines regularly and 33% used 10 or more medicines. The most commonly prescribed therapeutic subgroups were antithrombotic (70% of patients), non-opioid analgesics (58%), beta-blockers (47%), lipid-modifying drugs (41%) and hypnotics/sedatives (39%).  - Approximately one-fourth received potentially inappropriate medications, and over half was exposed to drug-drug interactions. Both PIMs and DDIs were positively correlated with the number of medicines prescribed and negatively associated with patient age. |
| Larsen *et al*. (2020) [27] | To explore whether exercise therapy and polypharmacy are associated with frailty state transitions for home care service recipients. | Alberta, British Columbia, Ontario, and Yukon, Canada | Longitudinal cohort study using client level health information collected using interRAI home care (RAI-HC) assessments | Not Reported | ≥9 medications | 250,428 | ≥65 | - 44.9% of participants were experiencing polypharmacy (≥9 medications). Non-frail clients using polypharmacy were more likely to become prefrail and frail. Pre-frail clients experiencing polypharmacy were more likely to become frail, and they were less likely to become non-frail. Frail clients experiencing polypharmacy were significantly less likely to become prefrail or non-frail.  - 80.4% of clients experiencing polypharmacy if defined as ≥5 medications. |
| Manis *et al.* (2020) [28] | To investigate the associations  between person-level characteristics and the rate of falls among home care clients, and to examine differences between males and females, and different high-risk subgroups | Ontario, Canada | Population-based, cross-sectional study | In Ontario, home care services are predominately provided by the provincial government under its universal, public health insurance plan to support older adults in receiving the care services they need (e.g., nursing, physiotherapy, occupational therapy, social work, etc.) to remain in their home and community. | Not Reported | 10,586 | 9% <60, 12% 60-69, 22% 70-79, 57.5% >80 | - Polypharmacy and health conditions had statistically significant associations with the rate of falls. Home care clients who took eight or more drugs had a 21% increase in the rate of falls.  - Home care clients with parkinsonism who took eight or more drugs had a 177% increase in the rate of falls, compared to those who do not have parkinsonism.  - 22% of home care clients were using 5-7 medications, and 64% were using 8 or more medications.  - Males who used assistive devices had a higher rate of falls compared to females; however, males with neurological and cardiovascular health conditions had a decrease in the rate of falls compared to females. |
| Rönneikkö *et al.* (2018) [35] | To identify typical discharge diagnoses and their associations with patient characteristics among home care clients who were hospitalised within one year of their first home care assessment. | Finland | A register-based study based on Resident Assessment Instrument-Home Care (RAI−HC) assessments | Not Reported | ≥5 medications | 6812 | ≥63 | - Polypharmacy was associated with a decreased probability of dementia Related hospitalisation.  - Polypharmacy was associated with and increased probability of hospitalisation due to cardiovascular reasons, however, polypharmacy was not associated with injuries.  - 42.0% of participants were experiencing polypharmacy, and 47.2% were experiencing excessive polypharmacy (10 or more medications)  - The most common reason for the first hospitalisation was an infectious disease (21%; n=1446) |
| Schneider *et al.* (2021) [44] | To measure the extent of polypharmacy, multimorbidity and potential medication  related problems in elderly patients with chronic pain receiving home care. | Berlin, Germany | A planned pre-specified sub-analysis of the ACHE study (observational cross-sectional analysis study) | Not Reported | ≥5 medications | 355 | ≥65 | - The prevalence of polypharmacy (≥5 prescribed drugs) was 89.5%(n 316) and almost half of the patients (n 174; 49.3%) were affected by excessive polypharmacy (≥10 prescribed drugs).  - There were no sex-specific differences for the prevalence of either polypharmacy or excessive polypharmacy.  - Patients affected by prescribed polypharmacy had significantly higher CCI (Charlson-Comorbidity Index) scores than patients without polypharmacy.  - 184 potentially relevant drug interactions in 34.0 % of patients were detected, and 31.0% of those were deemed severe.  - Under-prescription of oral anticoagulants was detected in 32.3% of patients with atrial fibrillation whereas potential overprescription of loop diuretics was observed in 15.5% of patients. |
| Sears *et al. (*2016) [29] | To explore medication knowledge and ability to take medication among seniors admitted to home care. | Ontario, Canada | Retrospective study | Home care provides various types of healthcare delivered at home to recovering disabled or chronically ill patients  who require medical, nursing, social, and therapeutic treatment and assistance with  activities of daily living. | Not Reported | 14,004 | ≥65 | - About ten percent had little or no knowledge of what medication to take (n=1,389/14,004) or an understanding of the  purpose of their medications (n = 1,396/ 14,004).  - Increasing numbers of medications prescribed was associated with decreased knowledge of medications.  - The strongest predictor of limited knowledge and ability to take medication was dementia.  - 11.9% of participants were taking 0-5 medications, 27.2% were taking 6-10, 28.3% were taking 11-15, and 32.6% were taking 16 or more medications. |
| Sino *et al.* (2013) [41] | To determine the medication management capacity of independently living older people on polypharmacy in relation to their cognitive- and self-management skills. | Two home care organisations in Netherlands | Cross-sectional study with a sample of older people receiving home care services experiencing polypharmacy | Not Reported | ≥ 5 medications | 95 | ≥75 years | - 48.4% (n= 46) of the participants were able to manage their medication by themselves at home.  - About 40% of the participants were unable to state the names of their medications, even with the aid of a medication list, and about 25% reported  having problems with opening medication packages.  - Self-management ability and medication management support were significantly associated with medication management capacity.  - Mean number of medications in participants was 9.3 (range= 5-18). |
| Sino *et al.* (2013) [40] | To determine whether home care workers can detect signs and symptoms indicative of potential ADRs in home care patients, using a standardised observation list. | Two home care organisations in Netherlands | Observational study | Home care workers help patients to live independently as long as possible, given the limits of their patients’ medical condition, by providing a range of services, such as help with bathing and getting dressed or intravenous therapy and injections, wound care, education on disease treatment, or assistance with medication intake. | Not Reported | 115 | ≥45 | - Mean number of prescribed medications was 8.8, and the mean number of over-the-counter medications was 0.7.  - In total, 234 signs and symptoms indicative of possible ADRs were observed by the home care workers, 116 (49.6%) of which were considered as drug related.  - 17 out of 20 observed falls could be drug related.  - Observed dizziness (64.1%) and drowsiness (53.3%) could be drug related in most cases.  - The experts considered that a high proportion of the fainting spells (71.4%), potentially indicative of renal or heart failure, could be drug related. |
| Sun *et al.* (2017) [30] | To evaluate the relationship between therapeutic self-care, adverse events, and health system  utilisation; while controlling for client demographics and clinical characteristics. | Ontario, Canada | A retrospective cohort study | Not Reported | ≥ 9 medications | 1470 | ≥18 | - The low self-care individuals were characterised as having more complexity in clinical status such as having recent hospitalisations; multiple chronic diseases, polypharmacy; physical symptoms such as oedema; and higher CHESS (Changes in Health, End-Stage Disease, Signs and Symptoms) scores.  - High polypharmacy was associated with the increased odds of experiencing new hospital visits among the individuals with low therapeutic self-care ability. Sixty-one percent of home care clients with polypharmacy were found to have unplanned hospitalisations in this study cohort. |
| Tiihonen *et al.* (2016) [36] | To compare discrepancies between in-home interviews and electronic medical records (EMRs) on regularly used prescription drugs among older home care clients. | Finland | Clinical trials, Identifier:  NCT02214758 | Not Reported | Excessive polypharmacy: ≥10 | 276 | ≥75, Mean: 84.5 | - Living with a spouse or other family member, use of private health care services, diagnosed asthma/COPD or excessive polypharmacy was associated with having discrepancies.  - 54.9 % of the home care clients were experiencing excessive polypharmacy.  - The most common clinically important discrepancies were psychotropics, opioids and agents acting on the renin–angiotensin system (ACE inhibitors and ARBs) and beta-blocking agents. |
| Vetrano *et al.* (2014) (abstract only) [37] | To describe the co-occurrence of specific illnesses and geriatric conditions in a sample of older adults receiving home care services. | Czech Republic, Denmark, Finland, France, Germany, Iceland, Italy, Netherlands, Norway, United-Kingdom, Sweden | Retrospective cross-sectional study | Not Reported | ≥5 medications | 4007 | Mean: 79 | - The most common geriatric conditions were pain (81%), incontinence (46%), and polypharmacy (38%).  - Participants with cardiovascular diseases presented the highest level of multimorbidity (78–80% with ≥3 diseases). Those affected by dementia had the lowest (48% with ≥3 diseases). Dementia and Parkinson’s disease were associated with the highest number of Geriatric conditions.  - Study participants presented also on average 4.1 geriatric conditions. The most common diseases were hypertension (33%), osteoarthritis (26%) and heart failure (24%). |
| Wang-Hansen *et al.* (2019) [43] | To explore risk factors of serious adverse drug events (SADE) and SADE-related admissions, and assess whether these could have been prevented by adherence to  the prescription tools Screening Tool of Older Persons’ Prescriptions (STOPP) and The Norwegian General Practice (NORGEP) criteria. | Norway | Cross-sectional study | Not Reported | 5–9 regular drugs, excessive polypharmacy:  ≥10 regular drugs | 232 | ≥75, mean: 86 | - 52% of participants used 5-9 drugs whereas 28% used ≥10.  - SADEs were identified in 31% of the patients, and in 68% of these cases, the SADE was considered to cause the hospital admission.  - Among the SADEs identified, 44% and 15% were preventable by adherence to STOPP and NORGEP, respectively.  - According to the NORGEP criteria, the most commonly used inappropriate drug combinations causing SADEs were warfarin in combination with NSAIDS or SSRI and the use of three or more drugs from the groups centrally acting analgesics, antipsychotics, antidepressants, and/or benzodiazepines.  - The most frequent SADE was anaemia or gastrointestinal bleeding caused by antithrombotic agents alone or in various combinations with interacting drugs such as non-steroidal inflammatory drugs (NSAIDs), glucocorticoids, and selective serotonin reuptake inhibitors (SSRI). Hypokalaemia and  hyponatremia induced by diuretics and drugs acting on the renin-aldosterone-angiotensin system was the second most frequent present SADE. |
| Key: RAI-HC = Resident Assessment Instrument for Home Care; ADLs = Activities of Daily Living; OTC = Over-the-Counter; PIMs = Potentially Inappropriate Medications; AE = adverse events; MDD = Multidose Drug Dispensing; DDIs = Drug-Drug Interactions; CCI = Charlson-Comorbidity Index; ADRs = Adverse Drug Reactions; CHESS scores = Changes in Health, End-Stage Disease, Signs and Symptoms scores; EMRs = Electronic Medical Records; SADE = Serious Adverse Drug Events; STOPP criteria = Screening Tool of Older Persons’ Prescriptions criteria; NORGEP criteria = The Norwegian General Practice criteria. | | | | | | | | |
